# NTLA-2001: opening a new era for gene therapy

**DOI:** 10.1093/lifemedi/lnac036

**Published:** 2022-08-30

**Authors:** Tongtong Cui, Bojin Li, Wei Li

**Affiliations:** State Key Laboratory of Stem Cell and Reproductive Biology, Institute of Zoology, Chinese Academy of Sciences, Beijing 100101, China; Institute for Stem Cell and Regenerative Medicine, Chinese Academy of Sciences, Beijing 100101, China; Beijing Institute for Stem Cell and Regenerative Medicine, Beijing 100101, China; State Key Laboratory of Stem Cell and Reproductive Biology, Institute of Zoology, Chinese Academy of Sciences, Beijing 100101, China; Institute for Stem Cell and Regenerative Medicine, Chinese Academy of Sciences, Beijing 100101, China; University of Chinese Academy of Sciences, Beijing 100049, China; State Key Laboratory of Stem Cell and Reproductive Biology, Institute of Zoology, Chinese Academy of Sciences, Beijing 100101, China; Institute for Stem Cell and Regenerative Medicine, Chinese Academy of Sciences, Beijing 100101, China; Beijing Institute for Stem Cell and Regenerative Medicine, Beijing 100101, China; University of Chinese Academy of Sciences, Beijing 100049, China

Recently, Intellia Therapeutics, a gene-editing company co-founded by Nobel laureate Jennifer Doudna, along with Regeneron Pharmaceuticals, created history and reported the first clinical proof for direct CRISPR genome editing in humans to treat transthyretin amyloidosis (ATTR), a genetic disease afflicting around 50,000 people worldwide, by therapeutically rewriting the faulty genomes of patients. The work presenting this CRISPR-based gene therapy, NTLA-2001, was published in The New England Journal of Medicine [1]. Clinical results have shown that upon a one-off intravenous infusion, patients receiving this CRISPR-based genetic treatment produced far fewer harmful misfolded transthyretin (TTR) proteins, which would progressively threaten their lives (ClinicalTrials.gov number, NCT04601051). More excitingly, the latest data released showed that NTLA-2001 treatment at two different doses achieved mean reductions of 86% and 93% in serum TTR levels by day 28. And long-lasting effects of CRISPR infusion were observed (Intellia Therapeutics Press Release).

ATTR, manifested as the accumulation of misfolded TTR proteins, is a progressive and life-threatening disease. Hereditary ATTR (hATTR) is caused by structurally abnormal TTR proteins due to >100 different mutations in *TTR*. The amyloid deposits accumulate in multiple tissues, predominantly the heart, nerves, and gastrointestinal tract, leading to polyneuropathy, and cardiomyopathy. Most patients have a life expectancy of from 2 to 15 years. Current treatment strategies include silencing *TTR* mRNA by inotersen or patisiran and stabilizing TTR tetramers by tafamidis [2]. Nevertheless, these methods have limitations, especially like the requirement for long-term repeated administration and unavoidable disease progression. Hence, development of a one-time effective hATTR treatment to overcome these drawbacks is in urgent need. Directly editing the *TTR* in hepatocytes using the CRISPR-Cas nuclease became an appealing option.

**Figure 1.**
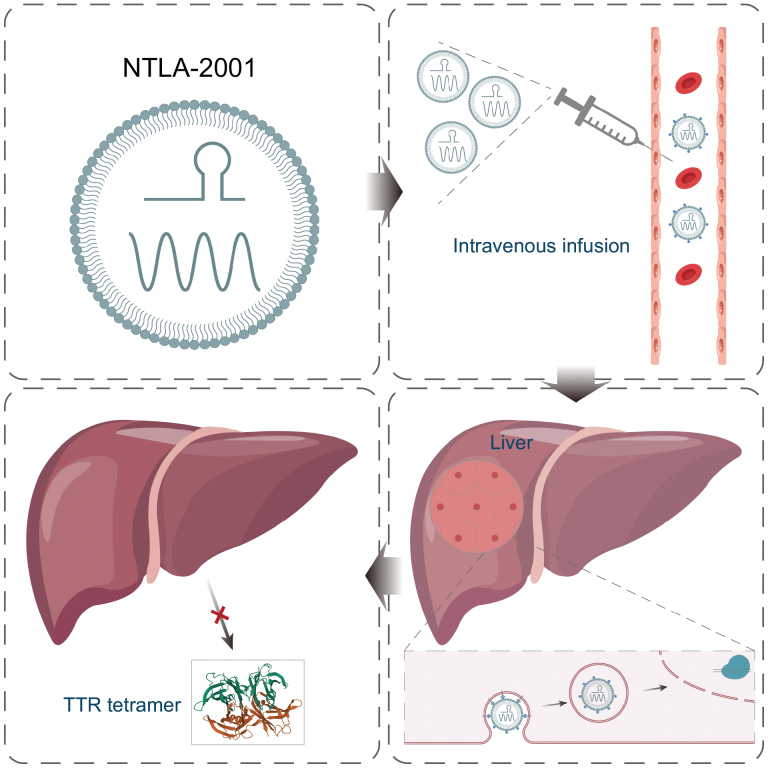
A schematic overview of NTLA-2001 gene therapy. NTLA-2001 comprises LNPs, sgRNAs targeting *TTR*, and mRNAs for Cas9. Intravenous infusion is followed by targeted delivery of CRISPR components to the liver, where the knock-out of *TTR* takes place, leading to a reduced TTR protein level in human serum.

The discovery of CRISPR-Cas nucleases fueled the research of *in vivo* gene-editing therapies since they can directly modify genomic DNA at the endogenous locus and ablate the pathogenic mutation with high specificity. The quick turnaround time achievable between design and validation of new gRNAs combined with the high efficiency to induce double-strand breaks enabled the fast adoption of CRISPR-Cas9 by a wide swath of researchers. Meanwhile, different types of CRISPR-Cas systems have been discovered and adapted for research [3]. In less than a decade since its emergence, this powerful tool has been developed into various therapeutic agents for illnesses including β-mediterraneanb anemia, LCA10, and hemoglobinopathy. Here, this study introduced a new approach, NTLA-2001, using Intellia’s proprietary non-viral platform that deploys lipid nanoparticles (LNPs) to deliver a two-part CRISPR-Cas9 editing system to permanently knock out human *TTR*, and eventually reduce TTR accumulation (Fig. 1).

NTLA-2001 exhibited positive interim results in phase 1 clinical trials [1]. It achieved high-level serum TTR reductions in six hATTR patients associated with efficient *TTR* knock-out after a single dose administration. The patients were divided into two groups of three, each intravenously infused with NTLA-2001 at a dose of 0.1 and 0.3 mg/kg, respectively. All six patients exhibited a noticeable decline in serum TTR levels 28 days after treatment: the lower dose cohort showed reductions ranging from 47% to 56%, while the higher dose cohort had reductions ranging from 80% to 96%. This result was already comparable to or more promising than available standard therapies for ATTR-triggered polyneuropathy, which are usually associated with a reduction of around 80%. More importantly, only mild adverse events occurred.

The improvement was significant, and extended follow-up information from fifteen patients receiving NTLA-2001 treatment in four single-ascending dose cohorts continues to be reported. According to the most recent data (Intellia Therapeutics Press Release), therapeutic effects of NTLA-2001 are long-lasting. The follow-up time with these patients reached 12 months at most, and all individuals showed a sustained reduction of TTR proteins. Meanwhile, at day 28, the mean reduction from baseline in serum TTR protein concentration were 86% and as much as 93% in other nine patients who received higher doses at 0.7 and 1 mg/kg, respectively. Overall, dose-dependent effects were observed. These results support the therapy’s continued development.

The sustained durability of significant TTR reduction in patients was actually expected based on earlier preclinical tests in mice and non-human primates. In the previous studies, Finn *et al.* and Wood *et al.* have provided the roadmap for the preclinical development of this gene-editing therapeutic by describing the proof of concept, pharmacology, and comprehensive specificity as well as tolerability results. Finn *et al.* reported that an LNP-delivered Cas9 nuclease mRNA and a mouse *TTR*-targeting sgRNA successfully disrupted *TTR* in the liver and led to a substantial and durable reduction in TTR protein levels [4]. Then Wood *et al.* acquired similar results using cynomolgus monkeys [5]. To conclude, there was an NTLA-2001 dose-dependent increase in liver gene-editing efficiency observed in both transgenic mice and non-human primates, where a high dose led to a nearly complete reduction of serum TTR protein level. Particularly, in cynomolgus monkeys, a single dose at 3.0 mg/kg led to durable reductions in serum TTR protein of >94%.

As gene-editing efficiency may change remarkably when moving the context from immortalized cell lines to *in vivo* environment due to a variety of reasons, it is critical to choose an appropriate delivery system. In gene-therapy studies, the therapeutic cargos are usually carried by viral systems, AAVs in most cases, which may lead to immune responses. The LNP is currently the lead non-viral delivery system for genetic drugs due to its transient, non-integrating, and immune response-limited nature. Meanwhile, it has been proven to be a solid delivery system with liver tropism for RNAs. Here, NTLA-2001 utilized LNPs to carry sgRNAs targeting *TTR* and mRNAs of Cas9 proteins, which helps maximize efficacy while minimizing systemic toxic effects [1].

Safety has long been a primary concern in gene-therapy research. It is encouraging that this NTLA-2001 project has put efforts to address safety issues well at this stage. As for experimental proof, a high therapeutic index (i.e. the ratio of on-target to off-target editing) was selected by performing genome-wide assays and targeted sequencing to identify and verify candidate sgRNA off-target sites. Possible off-target sites identified through Cas-OFFinder, GUIDE-seq, and SITE-seq were all located in noncoding regions, and no evidence of off-target editing was found when primary human hepatocytes were treated with high levels of NTLA-2001 [1]. Moreover, the most recent clinical data shows that after NTLA-2001 treatment for up to 12 months, it was generally well tolerated among 15 patients receiving different doses of up to 1 mg/kg. Only one possibly related severe adverse event of vomiting (Grade 3) was reported in the 1 mg/kg group, while mild adverse events (Grade 1) accounted for the most (Intellia Therapeutics Press Release).

Based on all the previous interim data, it is becoming more apparent that NTLA-2001 serves as a promising treatment for patients with ATTR amyloidosis. The safety, depth, and durability of serum TTR reduction achieved by NTLA-2001 underscore its enormous potential to stop and even reverse this disease after a single-dose treatment. This is a great advance toward genetic medicine. Since the concept of gene therapy arose in the 1970s, the application of the enormous genomic knowledge we have gained during the past several decades to prevent, diagnose, and treat human disease is holding great promise for revolutionizing medical care. In 2003, China approved the world’s first commercially available gene therapy GENDICINE to treat head and neck squamous cell carcinoma. As of 20 July 2022, Upstaza, the first gene therapy directly infused into the brain, was approved by EMA, making it another momentous event not only for AADC deficiency patients but for the entire gene therapy community.

As the interim results have met a statistically significant and clinically meaningful change from the baseline for serum TTR reduction, all of us are looking forward to the progress of this clinical development of the first-ever systemically administered *in vivo* CRISPR therapy. We expect that NTLA-2001 will lead the long-awaited CRISPR-based gene therapy down the road to success.
